# MGEs as the MVPs of Partner Quality Variation in Legume-Rhizobium Symbiosis

**DOI:** 10.1128/mbio.00888-22

**Published:** 2022-06-27

**Authors:** Katy D. Heath, Rebecca T. Batstone, Mario Cerón Romero, John G. McMullen

**Affiliations:** a Department of Plant Biology, University of Illinois at Urbana-Champaign, Urbana-Champaign, Illinois; b Institute for Genomic Biology, University of Illinois at Urbana-Champaign, Urbana-Champaign, Illinois; c Department of Biology, Indiana University, Bloomington, Indiana

**Keywords:** ICE, MGEs, evolution, mutualism, plasmid, symbiosis

## Abstract

Despite decades of research, we are only just beginning to understand the forces maintaining variation in the nitrogen-fixing symbiosis between rhizobial bacteria and leguminous plants. In their recent work, Alexandra Weisberg and colleagues use genomics to document the breadth of mobile element diversity that carries the symbiosis genes of *Bradyrhizobium* in natural populations. Studying rhizobia from the perspective of their mobile genetic elements, which have their own transmission modes and fitness interests, reveals novel mechanisms for the generation and maintenance of diversity in natural populations of these ecologically and economically important mutualisms.

## COMMENTARY

We are witnessing a sea change in the way evolutionary biologists think and write about the mutualism between rhizobia and their legume hosts. While variation in these large and complex bacterial genomes and its impact on plant growth and fitness has been of interest for a long time, the “mobile genetic element’s (MGE)-eye view” (2) is making its mark on the questions we ask about mutualist partner quality, genome evolution, and geographic variation. Of course, the mobile nature of symbiosis genes in rhizobia is not particularly new (3, 4), and evolutionary biologists like Jennifer Wernegreen recognized that symbiosis genes can have their own evolutionary history decades ago (5, 6). However, as microbial genomes abound, a powerful integration is beginning to emerge between studies of how selection (host-mediated and otherwise) operates on the genetic variation in natural populations and the mechanisms of mobility and their implications for the processes that generate, move, and maintain that genetic variation. The recent collaboration exemplified in Weisberg et al. (1) takes a population genomics approach to the MGE landscape underlying rhizobial partner quality to reveal how nestedness, modularity, and the larger community context of symbiosis MGEs can contribute to the maintenance of mutualistic variation in nature.

## NESTEDNESS

**FIG 1:**
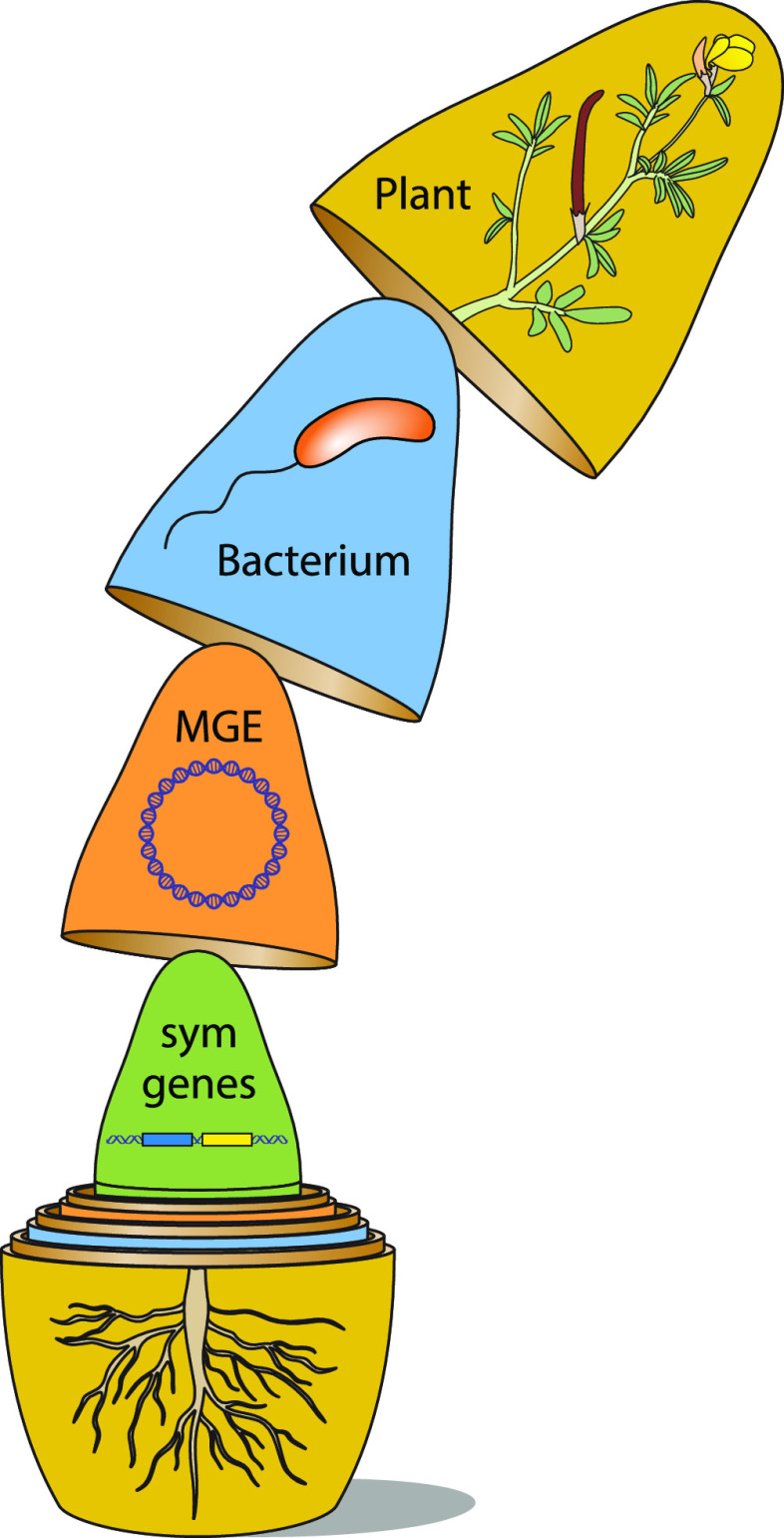
Levels of horizontal mobility lead to nested interactions among the key players in the symbiosis between leguminous plants, nodulating rhizobial bacteria, the mobile genetic elements (MGEs) they host, and the symbiosis (sym) genes often carried on those MGEs.

The results of Weisberg et al. (1) highlight the nested structure of *Bradyrhizobium* genomes: symbiosis (sym) genes embedded within sym integrative and conjugative elements (symICEs), which in turn are embedded within *Bradyrhizobium* chromosomes. Importantly, sym genes can be shuffled across different ICE (or even plasmid!) backbones via recombination, while ICEs are transferred among *Bradyrhizobium* strains via excision and interbacterial conjugation. When in symbiosis, sym genes reside on and interact with these MGEs (e.g., ICEs, plasmids), which interact with bacterial chromosomes in the bacterial cell, all of which reside within the symbiotic nodules on plant roots, resulting in a series of nested interactions reminiscent of a Ukrainian nesting doll (Fig. 1). Because each nested scale exhibits some degree of mobility enabled by horizontal transmission, each component exhibits its own evolutionary history that is distinct from that of the genomic background in which it temporarily resides. For example, the multilocus phylogenetic tree based on housekeeping genes in *Bradyrhizobium* chromosomes has a completely different branching pattern than the *nodA* locus located on various ICE subtypes (1). Mobility thus bestows upon each nested scale its own evolutionary agency—or the drive to survive and replicate—and thus, each scale represents a “genetic individual” upon which selection can act independently from the genomic background (7, 8).

Through this lens, we should consider *Bradyrhizobium* ICEs (and other rhizobial MGEs) to be evolving in a complex ensemble of nested genomic populations rather than in a vacuum: populations of MGEs evolve within the context of populations of bacterial chromosomes, whereby the population size, mutation rates, and even geographic structure of one population does not necessarily reflect the structure of the other (9–11). Barriers to gene flow might be present at one scale and absent at another, and coevolution might occur among just some of the nested scales. Given the strong effects of sym genes on the growth and fitness of both bacteria and hosts (12), signatures of coevolution might be most apparent among sym genes and plant loci, while less-mobile loci within rhizobial chromosomes (e.g., housekeeping genes) might be like middlemen, bystanders in the coevolutionary dialogue between plant and sym loci and subject to distinct selective pressures (13, 14). Together, the nested genomic interactions in legume-rhizobium symbiosis likely mean that the evolution of the traits we most care about (N-fixation efficiency, plant growth, and plant nutrient status) cannot be predicted solely by additive effects of loci across nested scales, given that these traits are determined by a multitude of nested and interacting genomes.

## MODULARITY AND DIVERSITY

Weisberg et al. (1) describe a process wherein the flexibility of a modular mechanism both maintains the function of nitrogen (N) fixation and promotes genetic variation in *Bradyrhizobium*. This presents a seeming paradox in which less-beneficial partnerships are generated by the process of creating genetic variation and robustly maintaining N fixation. However, this paradox might be resolved with a holistic MGE-eye’s view of the potential selection in both free-living and symbiotic environments in a nested system with MGE agency, and should be applicable to other rhizobium-legume systems, including *Mesorhizobium*, which also has symICEs. Strains of *Mesorhizobium* sampled from chickpea crops around the globe form populations determined by biogeographic patterns, but their sym genes have a more extended geographic range than the bacterial host chromosomes (15). Likewise, there is evidence of uncooperative mutants (without symICEs) frequently co-occurring with cooperative kin in wild populations of *Mesorhizobium* (16), again indicative of symICEs with distinct MGE population dynamics and fitness maintaining genetic variation for legume-rhizobium symbiosis. We will benefit from comparing how modularity and diversity generate and maintain variation in *Rhizobium* and *Ensifer*, two lineages where N fixation genes are primarily placed on plasmids (17, 18), which implies a few differences in the evolutionary dynamics of their mechanism for N fixation. The fact that the population structure and phylogenies of sym genes tend to disagree with those of housekeeping or chromosomal genes (11, 19–21) is evidence of the modularity of this mechanism. But conclusive evidence on how natural selection acts at the various nested levels in the wild, and across the diverse selective environments in which rhizobia find themselves, is needed to quantify how MGE flexibility and modularity generates both variation and robustness.

## COMMUNITY CONTEXT

Finally, Weisberg’s MGE-eye’s view helps reveal how less-beneficial rhizobia on a focal plant species might be maintained in part through effective symbiosis with alternative host species. Although we often study this symbiosis as a pairwise interaction in experimental work, rhizobia and their legume hosts do not exist in a vacuum, devoid of other species and interactions in nature. Indeed, rhizobia exist in more complex microbial communities while in their soil-dwelling phase (22), including other species of rhizobia (23, 24). Natural plant communities are not monocultures, and often include additional species of legumes that could serve as potential partners (25, 26). Assemblages of sympatric plant and microbial species provide environmental heterogeneity that has the potential to favor different plant-bacterial-MGE-sym gene combinations (Fig. 1). Selection might even drive mobilization of key sym genes among strains or even species of rhizobia (27), especially when host distributions are patchy (2). Weisberg and colleagues (1) identify several ineffective strains and test whether they may infect other sympatric species and provide comparable beneficial effects to these alternative hosts (1). Most of the ineffective symbiont strains on the focal host plant (California native *Acmispon strigosus*) could infect the roots of other potential hosts, though only a single strain displayed a beneficial effect. This strain largely contained the core genes required for effective symbiosis but had a varied organization of the associated symbiosis MGEs compared to others of its subtype (1). Together these observations suggest that sym gene modularity and flexibility can generate benefits for co-occurring legume hosts. Similarly, community-level interactions among rhizobia and multiple hosts might contribute to the coexistence of sympatric species of clover found in natural populations in Northern California (28). Thus, understanding the maintenance of genetic variation within a single legume-rhizobium symbiosis will require that we integrate the larger community context of rhizobia and hosts alongside the more commonly considered factors such as abiotic conditions (29–31), intraspecific genetic diversity and G x E (32, 33), and soil selection (14, 34).

## CONCLUSION

As we continue to make sense of how legumes and rhizobia evolve in the context of diverse and complex natural ecosystems, a synthesis of the MGE-eye’s view across systems will be critical. Together with ecological genetic work focused on measuring natural selection and phenotypic variation as well as increasingly community-aware perspectives on evolution, this synthesis will move us toward a predictive understanding of how N-fixing symbiosis evolves in the wild and improve our ability to manage symbiotic outcomes toward conservation and sustainable agriculture goals.
